# Interferon Lambda Genetics and Biology in Regulation of Viral Control

**DOI:** 10.3389/fimmu.2017.01707

**Published:** 2017-12-06

**Authors:** Emily A. Hemann, Michael Gale, Ram Savan

**Affiliations:** ^1^Department of Immunology, Center for Innate Immunity and Immune Diseases, University of Washington, Seattle, WA, United States

**Keywords:** interferon lambda, interferon, immunity, immune cells, infectious disease, virus

## Abstract

Type III interferons, also known as interferon lambdas (IFNλs), are the most recent addition to the IFN family following their discovery in 2003. Initially, IFNλ was demonstrated to induce expression of interferon-stimulated genes and exert antiviral properties in a similar manner to type I IFNs. However, while IFNλ has been described to have largely overlapping expression and function with type I IFNs, it has become increasingly clear that type III IFNs also have distinct functions from type I IFNs. In contrast to type I IFNs, whose receptor is ubiquitously expressed, type III IFNs signal and function largely at barrier epithelial surfaces, such as the respiratory and gastrointestinal tracts, as well as the blood–brain barrier. In further support of unique functions for type III IFNs, single nucleotide polymorphisms in *IFNL* genes in humans are strongly associated with outcomes to viral infection. These biological linkages have also been more directly supported by studies in mice highlighting roles of IFNλ in promoting antiviral immune responses. In this review, we discuss the current understanding of type III IFNs, and how their functions are similar to, and different from, type I IFN in various immune cell subtypes and viral infections.

## Evolution of Type III IFN Genes

Type I IFN is produced and secreted rapidly following viral infection (1, 2). It subsequently signals to surrounding cells to initiate an antiviral state as a critical host defense mechanism. In humans, there are 13 subtypes of IFNα as well as IFNβ, IFNε, IFNκ, and IFNω [reviewed in Ref. (3)]. Type I IFNs are intronless genes clustered on chromosome 9 in humans and chromosome 4 in mice. In mammals, birds, reptiles, and amphibians, type I IFN genes lack introns, which suggests their origin may have been from retrotransposed genetic elements [reviewed in Ref. (4)]. However, type I IFNs in fish harbor introns and are thought to have arisen through a common ancestor of IL-10 family [reviewed in Ref. (5)]. Amphibians have been recently described to have both intron-containing and intron-less type I IFN genes (6). The current understanding of interferon evolution has not distinguished whether an independent or retrotransposition event led to the generation of intron-less type I IFN genes that may have been the ancestor of the intron-less type I IFN locus in reptiles, birds, and fish.

IFN lambda family members were initially named as interleukin-28 (IL-28) and IL-29 and classified into the IL-10 family genes as they signal through the common IL-10 receptor subunit 2 (IL-10R2) (7, 8). Humans have four *IFNL* genes, *IFNL1* (*IL29*), *IFNL2* (*IL28A*), *IFNL3* (*IL28B*), and *IFNL4*. *IFNL* genes are present in tetrapods, but in contrast to the evolutionary diversity seen in type I IFNs, throughout vertebrates the type III IFN locus comprised of two to four family members, each containing introns (9). While IFN lambdas are most functionally similar to type I IFNs, they are structurally similar to members of the IL-10 family. Type III IFNs have a phase 0 intron–exon structure and utilize a component of IL-10R2 as a part of their receptor heterodimer complex for signaling (10). Sequence identities of type III IFNs when compared with type I IFNs (15–19% aa) or IL-10 (11–13% aa) are low (8). Among type III genes, *IFNL1* and *IFNL2* share 81% amino acid identity, whereas *IFNL2* and *IFNL3* share 96% amino acid identities. *IFNL4* shares only ~28% amino acid identity with other *IFNL* genes, leading to speculation *IFNL4* may have been introduced *via* a separate duplication event. While the evolutionary history of type III IFNs is still incomplete, a number of groups are working to understand the evolutionary constraints on type III IFNs [reviewed in Ref. (4, 11, 12)]. Utilizing an evolutionary genetics approach, Manry et al. demonstrated that type I and type III IFNs, and even individual genes within each of these types, have been subjected to distinct evolutionary pressures (11). This work suggests both redundant and specific, unique roles for these IFN families in pathogen defense.

In contrast to humans, in mice only *Ifnl2* and *Ifnl3* are functional; *Ifnl1* and *Ifnl4* are pseudogenes (13). Despite differences in human and murine *Ifnl* gene composition, murine studies have provided critical insights into the antiviral and immune modulatory functions that have relevant correlates to human infection. For example, in a murine asthma model, interferon lambda (IFNλ) treatment was demonstrated to lead to a Th1-biased immune response (14). In humans, IFNλ leads to enhanced Th1 responses during influenza virus vaccination (15). In addition, respiratory viral pathogens have evolved mechanisms to suppress IFNλ function or downstream signaling, highlighting the critical importance of IFNλ to respiratory immunity in particular, but also the contribution of IFNλ to infection at mucosal barriers in general (16, 17).

## Expression IFN Lambda Genes During Viral Infection

IFNs are expressed following detection of pathogen-associated molecular patterns (PAMPs) by pattern-recognition receptors (PRRs) [reviewed in Ref. (18–20)]. Sensing of PAMPs by the RIG-I-like receptors results in the recruitment of mitochondrial antiviral signaling protein (MAVS) to mitochondrial associated membranes or peroxisomes, leading to activation of the transcription factors NF-κB and interferon regulatory factors (IRFs), which induce expression of both type I IFN and IFNλ (21). Multiple toll-like receptors induce expression of type I and III IFNs (22, 23). While the signals and pathways that induce type I and type III IFNs largely overlap, one notable exception does exist in the DNA sensing pathway. In HEK293 and THP-1 cells, binding of DNA to the cytosolic sensor Ku70 induces production of IFλ1 and IFNλ2/3 but not type I IFN (24). Following transfection of DNA or herpes simplex virus-2 infection, DNA binding to Ku70 leads to recruitment of STING and subsequent activation of IRF3 in addition to IRF1 and IRF7 (24, 25). Whether this novel, IFNλ-specific IFN induction exists in other cell types following DNA sensing is an interesting possibility that has not yet been investigated.

Although type I and III IFNs are all induced following infection, the transcription of these genes is temporally regulated. Type I IFNs are induced and resolved rapidly, followed by a delayed but sustained induction of *IFNL* genes (19, 26, 27). The mechanisms responsible for a distinct temporal induction pattern of type I and type III IFNs is currently unknown, but this could be due to utilization of different signaling molecules or transcription factors. The *IFNL1* and *IFNL3* promoters harbor binding sites for IRF1, IRF3, IRF7, and NF-κB (28). However, in contrast to type I IFNs, studies have suggested that transcription of *IFNL* is primarily dependent on NF-κB, and activation of both IRF and NF-κB signals is required for a robust induction of *IFNL* (29). The differential requirement for IRFs and NF-κB in the induction type I and type III IFNs following PAMP engagement by the PRRs could potentially contribute to the temporal difference in their transcriptional regulation of type III IFNs compared with type I IFNs.

Both type I and type III IFNs are produced following rotavirus infection in an adult murine model, but intestinal epithelial cells (IECs) respond preferentially to type III IFN (27, 30), suggesting a predominant role for IFNλ in antiviral defense in the intestine. In addition, type III IFNs are produced more abundantly at mucosal sites by epithelial and myeloid cells in response to viral infection (31). The mechanism for this preferential induction of type III IFN by IECs remains to be fully elucidated, but it might be due in part to the preferential induction of IFNλ upon MAVS localization to peroxisomes, which are highly abundant in epithelial cells, following PAMP sensing (21). Another possible mechanism is that undefined tissue-specific factors present at the epithelial barrier surfaces may promote IFNλ over type I IFN, similar to the IFNλ response in hepatocytes during hepatitis B and hepatitis C virus (HCV) infection (32). Further, IFNλ can be induced by type I IFN similar to an interferon-stimulated gene (ISG) in a feed-forward fashion (33). This type I IFN enhancement of *IFNL* is at least partially due to the ability of type I IFN to increase TLR expression; however, the functional consequences of this co-regulation remain to be tested.

Overall, a lack of IFNλ-specific mouse models and antibody detection reagents for ligands and receptors has slowed progress in determining the contribution of IFNλ to immunity. While whole body knockout mice lacking IFNλR exist, dissection of the role IFNλ signaling in various tissues and cell types *in vivo* will be advanced by studies in mice utilizing a recently reported floxed IFNλR model (34). In addition, an IFNλ2 cytokine reporter mouse has recently been developed (35). These new models will likely lead to a rapid advancement in understanding the unique functions of IFNλ *in vivo*.

## IFN Lambda Receptor Expression and Signaling

**Figure 1:**
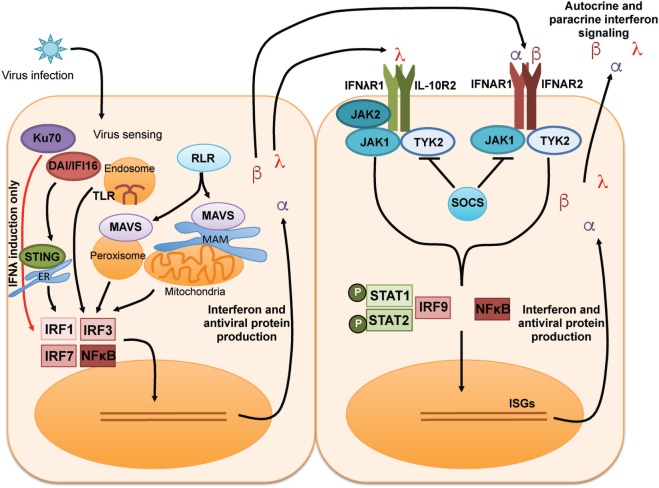
General induction and receptor signaling pathways of type I and type III IFNs. Recognition of virus by multiple pattern recognition receptor pathways leads to the activation of the transcription factors IRF1, IRF3, IRF7, and NF-κB to induce transcription, translation, and secretion of type I IFN (IFNα and IFNβ) and type III IFN [interferon lambda (IFNλ)]. Type I and type III IFNs signal to surrounding cells *via* distinct receptors to induce activation of the JAK–STAT pathway leading to the production of IFN-stimulated genes (ISGs) that can amplify the IFN signal and induce an antiviral state in infected cells/tissues.

The general induction and signaling cascades of type I and type III IFNs are summarized in Figure 1. Type I and III IFNs each signal through distinct receptor heterodimer complexes [reviewed in Ref. (3, 17, 19, 36)]. Type I IFN binds to a receptor complex comprised of IFNAR1 and IFNAR2, which is broadly expressed on most cells [reviewed in Ref. (1, 2)]. IFNλ signals through a heterodimeric receptor comprised of IFNλR1 and IL-10R2 (7, 8); IL-10R2 is a receptor subunit that is broadly expressed and shared for signaling by members of the IL-10 cytokine family [reviewed in Ref. (37)]. By contrast, the expression of IFNλR1 is much more restricted to epithelial cells, subsets of myeloid cells, and neuronal cells. This limited expression likely explains the importance of IFNλ at mucosal sites and the blood/brain barrier [reviewed in Ref. (17, 38)]. Engagement of all IFNs with their receptors initiates downstream signaling events, namely, activation of the JAK–STAT signaling cascade. JAK1, TYK2, and potentially JAK2 are phosphorylated and activated, leading to subsequent phosphorylation and activation of STAT1 and STAT2, which then associate with IRF9. Together, the complex of STAT1, STAT2, and IRF9 is referred to as the interferon-stimulated gene factor 3 (ISGF3) transcriptional complex. Activated ISGF3 translocates to the nucleus and binds to the interferon-sensitive response element, initiating the transcription of a wide array of ISGs. SOCS1 can provide negative regulation of this JAK–STAT signaling pathway downstream of IFN *in vitro* and *in vivo* (39–41).

In addition to activation of the JAK–STAT pathway, IFNs also activate PI3K and MAPK signaling cascades (1, 2). Perhaps the shared utilization of these signaling pathways between IFN and many other cytokines may help to explain the varied role of IFN in modulating antiviral and immune responses in various contexts and locations. Different affinities for their respective receptors exist among IFN subtypes, which may alter the signal strength upon receptor engagement, thus potentially adding another layer of regulation in control of immune responses by IFNs. Mendoza et al. developed a high-affinity IFNλ3 to discern the structure of the cytokine. When used in *in vitro* experiments this high-affinity IFNλ3 was found to have enhanced HCV and hepatitis B virus (HBV) antiviral activity (42). These results support the idea that enhancing the strength of the interaction of IFN with its receptor can modulate downstream functions. While this particular study investigated antiviral and anti-proliferative responses, it would be interesting to discern whether engineering of high-affinity IFNλ molecules can alter other facets of immunity. The recently solved IFNλ3/IFNλR1/IL-10R2 signaling complex structure could aid in answering these questions and in the development of IFNλ therapeutic agonists that have differential affinities for the receptor complex and downstream signaling strengths (42). Other mechanisms to regulate the response to IFNλ at the level of the IFNλR are conceivable. For example, in addition to the restricted nature of the IFNλR1 subunit, a soluble, secreted IFNλR1 has been described that could potentially sequester IFNλ as a regulatory mechanism (43). In summary, further studies are needed to dissect the intricate interplay of how IFN signaling pathways function in concert with stimulation by other cytokines that may activate similar or overlapping intracellular signaling pathways.

## Antiviral Effects of Type III IFNs

Interferon lambda is important in a wide variety of viral infections that including HCV, HBV, influenza virus, rhinovirus, respiratory syncytial virus (RSV), lymphocytic choriomeningitis virus (LCMV), rotavirus, reovirus, norovirus, and West Nile virus (WNV) [reviewed in Ref. (17, 19, 44–47)]. Many of these studies of IFNλ antiviral responses have been focused on viruses that infect the liver, the respiratory, and gastrointestinal mucosa, and, more recently, those that cross the blood–brain barrier (BBB) to cause a neuroinvasive viral infection. Experimental *in vivo* approaches using IFNλR knockout mice have highlighted the importance of IFNλ signaling in control of influenza A virus (IAV), SARS coronavirus, RSV, and human metapneumovirus levels in the lung as well as norovirus, reovirus, and rotavirus levels in the gastrointestinal tract (30, 48–50). It is also of note that type I and type III IFNs also have roles in cancer, parasitic infections, fungal infections, and several bacterial infections that include potential respiratory pathogens such as *Staphylococcus aureus, Pseudomonas aeruginosa*, and *Mycobacterium tuberculosis*, as well as *Listeria monocytogenes* and *Salmonella typhimurium* in addition to IFNs regulation of viral infections [reviewed in Ref. (47, 51)]. As the contribution of type I and type III IFNs in these other settings has been recently reviewed, we will not elaborate further herein.

Multiple reports have suggested redundant roles for IFNα/β and IFNλ in response to infection (23, 28, 52). However, distinct contributions for IFNα/β and IFNλ to infection have begun to be appreciated. Table 1 summarizes viral infections where IFNλ has been demonstrated to contribute in comparison with the known role of IFNα/β in these infections *in vitro* and *in vivo*. While the differences between IFNλ and IFNα/β are still being investigated, studies have demonstrated the ISG response induced by IFNλ is reduced compared with IFNα/β, while *in vivo* IFNλ is much less inflammatory than IFNα/β (53–55). Interestingly, IFNλ retains many antiviral properties despite the less inflammatory response compared with type I IFNs. This has spurred development of IFNλ for clinical use as an alternative treatment to IFNα for HCV infection has been of recent interest (53). Enthusiasm within the HCV field for IFNλ as a therapeutic treatment has waned as a result of the availability of direct-acting antiviral drugs capable of clearing HCV infection (56). However, harnessing the potential antiviral and less inflammatory functions of IFNλ as a therapeutic may be useful in treatment of other hepatic viral infections.

**Table 1:** Interferon lambda (IFNλ) and IFNα/β functions in viral infection.

Virus infection	Role of IFNλ	Role of IFNα/β
**Negative-sense RNA viruses**		
Human metapneumovirus (−ssRNA Pneumoviridae)	IFNλ treatment reduces titer in murine model (57) Increased titers in mice lacking IFNλR and IFNAR (49)	Increased titers in mice lacking IFNλR and IFNAR (49) Increased titers and reduced CD8 T cell response in mice lacking IFNAR (58)

Influenza virus (−ssRNA Orthomyxoviridae)	Increased virus titers in human cells and murine models in the absence of IFNλR (48, 49) IFNλ reduced influenza A virus (IAV) titers with minimal-associated pulmonary damage in murine *in vivo* models (35, 48, 54, 55) Increased IFNλ [human single nucleotide polymorphism (SNP) rs8099917] correlates with increased Th1 skewing of CD4 T cell response and reduced sero-conversion following vaccination (15)	Mice lacking IFNAR1 and IFNλR in the stromal compartment are more susceptible to IAV infection (52) Therapeutic treatment of IAV-infected mice with IFNα leads to reduced IAV titers, but pulmonary damage (54)

Lymphocytic choriomeningitis virus (−ssRNA Arenaviridae)	IFNλ2 and IFNλ3 inhibit infection of human lung epithelial cells (59) IFNλR−/− mice have no change in virus titer, but increased CD8 T cell response to acute infection and reduced CD8 T cell response to chronic infection (60, 61)	Blockade of type I IFN controls persistent infection (62, 63)

Respiratory syncytial virus (−ssRNA Paramyxoviridae)	Increased titers in mice lacking IFNλR and IFNAR (49)	Increased titers in mice lacking IFNλR and IFNAR (49)

**Positive-sense RNA viruses**		
Dengue (+ssRNA Flaviviridae)	IFNλ1 induces expression of CCR7 and *in vitro* dendritic cell (DC) migration (64) IFNλ1 and IFNλ2 inhibit virus in a human epithelial cell line (65)	Mice lacking IFNAR are more susceptible to infection (66) Mice lacking IFNAR on CD11c+ or LysM+ cells have increased disease during infection, but still mount protective CD8 T cell responses against the virus (67)

Hepatitis C virus (HCV) (+ssRNA Flaviviridae)	SNPs rs4803217, rs8099917, rs12979860, and rs368234815 correlate with response to IFN therapeutic and spontaneous virus clearance (68–72)	IFNα therapeutic effective in control of HCV, but highly inflammatory (source)

Human immunodeficiency virus (+ssRNA Retroviridae)	IFNλ1, 2, 3 treatment of human monocyte-derived macrophages inhibits infection *via* JAK–STAT (73, 74) Pretreatment of human primary CD4 T cells with IFNλ1 or IFNλ2 reduced HIV integration and posttranscriptional events, but IFNλ1 was not negatively correlated with HIV levels *in vivo* (75)	Type I IFN can inhibit HIV *in vivo* in a humanized murine mouse model of infection (76) High, sustained type I IFN associated with pathogenicity during SIV infection of rhesus macaques (77) Serum IFNα inversely correlates with CD4 T cell counts in human patients with HIV-1 (78)

Norovirus (+ssRNA Caliciviridae)	Recombinant IFNλ clears persistent norovirus infection in a murine model, dependent upon IFNλR signaling in intestinal epithelial cells (IECs) (34, 50, 79) Mice lacking IFNλR have increased titers and virus shedding (50)	Persistence of norovirus in mice lacking IFNAR specifically on CD11c+ cells (80)

Rhinovirus (+ssRNA Picornaviridae)	IFNλ levels inversely correlate with rhinovirus replication in a human bronchial epithelial cell line (81)	Type I IFN response contributes to control of rhinovirus in murine airway cells at 37° (82)

SARS coronavirus (+ssRNA Coronaviridae)	IFNλR−/− mice have increased viral titers and shedding (49)	Type I IFN signaling in hematopoietic cells drives SARS-CoV pathogenesis in a murine model (83)

West Nile virus (+ssRNA Flavi)	Treatment with IFNλ protects mice from lethal infection IFNλR−/− mice have increased permeability of the blood–brain barrier and neuroinvastion of virus (84)	Mice lacking IFNAR have enhanced viral loads, increased tropism, and complete mortality (85)

Zika virus (+ssRNA Flaviviridae)	Knock down of IFNλR in HBMECs leads to increase in ZIKV dsRNA (86)	Mice lacking IFNAR susceptible to Zika virus infection (87) Zika virus antagonizes type I IFN response in human DCs (88)

**Double stranded RNA viruses**		
Reovirus (dsRNA Reoviridae)	Fatal disease in neonatal mice lacking IFNλR Mice lacking IFNλR fully or specifically in IECs have increased virus shedding and growth in IECs (34, 89)	No enhanced disease or systemic spread in IFNAR−/− mice infected intracranially (90)

Rotavirus (dsRNA Reoviridae)	IFNλ treatment (synergistically with IL-22) reduces rotavirus titer (91) Mice lacking IFNλR have increased virus titer (30)	Minimal role for IFNAR signaling in control of viral disease in mice (89)

**DNA viruses**		
Cytomegalovirus (dsDNA Herpesviridae)	IFNλ reduces replication and CD4 T cell proliferation in human PBMCs (92)	Type I IFN released by DCs inhibits replication (93) CMV directly inhibits type I IFN (94)

Hepatitis B virus (dsDNA Hepadnaviridae)	Restricts virus in murine cell line (32) Pegylated IFNλ augmented antiviral reduction in hepatitis B virus (HBV) levels of infected patients (95)	Type I IFN restricts HBV in hepatocytes (96) HBV inhibits type I IFN induction (97, 98)

Herpes simplex virus (HSV) (dsDNA Herpesviridae)	IFNλ inhibits HSV-1 and HSV-2 in human epithelial cells (99, 100) SNP rs12979860 correlates with HSV-1 severity upon reactivation (101)	INFAR−/− adult mice are susceptible to infection of the choroid plexus and HSV encephalitis, similar to newborn WT (102)

More recent and broad hypotheses posit that IFNλ treatment could also be utilized to control respiratory viral infections. In several experimental studies, prophylactic and therapeutic treatment of mice with IFNλ2 or IFNλ3 was shown to control IAV pulmonary titers similarly to IFNα or IFNβ treatment (54, 55). Importantly, IFNλ treatment avoided excessive pulmonary inflammation associated with IFNα treatment (54). The authors of this study speculated treatment with either cytokine overcame the known IAV NS1 mediated block on the induction of both type I and type III IFNs. The IFNλ treatment used in this study also altered responses in pulmonary monocytes and antigen presenting cells; however, the potential direct effects of IFNλ on these specific cell populations have not been characterized in an antiviral therapeutic setting.

## Genetic Association of IFN Lambda Locus to Viral Susceptibility

The function of *IFNL* genes and their ability to regulate immunity is further impacted by a number of single nucleotide polymorphisms (SNPs) that have been identified in genome-wide association studies and correlate strongly to infectious disease outcome. These have been described in great detail elsewhere [reviewed in Ref. (47)]. Here, we will briefly discuss more recent findings related to these SNPs where the mechanism of their function and direct outcome on immune responses has been described. There has been considerable progress in understanding the direct impact of these SNPs on immunity to infection and disease outside of correlative phenotypes.

Multiple SNPs in *IFNL3* are associated with response to interferon-based therapeutics and natural clearance of the HCV (68–72), although until recently the mechanism of regulation provided by these SNPs had not been understood. Our group has recently described the mechanism of one *IFNL3* SNP (rs4803217) where presence of the G allele correlates with HCV clearance, whereas the unfavorable T allele correlates with HCV persistence (103). Specifically, HCV was found to regulate expression of two microRNAs (miR-208b and miR-499a-5p) that target the 3′ untranslated region (UTR) of *IFNL3* leading to its degradation, allowing for viral persistence. The T allele leads to changes in the 3′ UTR allowing for enhanced binding of these HCV-induced microRNAs and AU-rich element-mediated decay of *IFNL3*, impacting expression of the cytokine and the outcome of HCV infection. Intriguingly, these same microRNAs also dampen type I IFN signaling in HCV-infected hepatocytes by downregulating expression of IFNAR1, a mechanism distinct from miR-208b and miR-499a-5p regulation of type III IFN (104).

Mechanistic studies have also defined the immunological consequence of another SNP impacting the production of IFNλ4. Approximately, 40% of Caucasians have an intact open reading frame for *IFNL4* gene (105). However, a frame-shift mutation (TT>dG at ss469415590) in *IFNL4* renders it a pseudogene. Intriguingly, the G gene variant encoding full-length *IFNL4* is strongly correlated with persistence of HCV. It was hypothesized, but not demonstrated, that *IFNL4* may have an intracellular role for dampening the antiviral response. However, it is speculated that this effect could be at least in part an indirect one as the dG *IFNL4* allele is linked with the less favorable *IFNL3* genotype at rs12979860 and rs4803217 (106). This work confirmed IFNλ4 has similar antiviral function to IFNλ3. However, the functional full-length *IFNL4* is induced at lower levels compared with *IFNL3* and is poorly translated due to intron-retention splice isoforms and weak polyadenylation (polyA) signal. Interestingly, non-human primates do not contain the dG>TT frame-shift mutation, but still limit IFNλ4 translation by production of intron-retention splice isoforms and a weak polyA signal, suggesting the functional IFNλ4 isoform has been selected against before the arise of the pseudogene frame-shift mutation in humans (106). It is still currently unclear as to why IFNL4 is suppressed, and perhaps undergoing pseudogenization. Perhaps *IFNL4* arose more recently through genetic duplication of *IFNL3* but did not develop a specific function distinct from IFNλ3, similar to what has occurred for other IFNs. Future studies without the confounding factor of linkage of the unfavorable *IFNL3* genotypes may reveal the function of bioactive IFNλ4 to antiviral immunity. In addition, more studies parsing out the mechanisms of *IFNL* SNPs regulation of disease could provide important insights for the development and functionality of IFNλ therapeutics.

## Type I VS III IFNs in Autoimmunity

The contribution of type I IFNs to development and manifestation of autoimmunity is well established [reviewed in Ref. (1, 107, 108)]. Type I IFNs are commonly upregulated in systemic autoimmune diseases such as systemic lupus erythematosus (SLE), Aicardi–Goutieres syndrome, Sjogren’s syndrome, type I diabetes, and psoriasis. More than half of adult patients, and 90% of pediatric patients, with SLE have elevated peripheral IFNα (109). Mechanistic studies have identified plasmacytoid dendritic cells (pDC), which are a major source of type I IFN, to be enriched in SLE lesions in humans and mice (110–112). Interestingly, type III IFNs do not seem to be linked to exacerbation of autoimmune diseases. In fact, type III IFN has been demonstrated to remediate symptoms in a mouse model of arthritis (113). Further, in a murine model of colitis, a disease that can be autoimmune in humans, IFNλ signaling specifically in neutrophils leads to a reduction on the release of reactive oxygen species and prevention of intestinal pathology (114). In addition, mice lacking the IFNλR1 have exacerbated disease in a model of asthma (14). This potentially protective role of IFNλ in asthma has also begun to be explored in humans (81). One recent paper has identified a correlation between systemic sclerosis and elevated IFNλ1 levels (115), but mechanistic studies clearly identifying a role for IFNλ in autoimmune disease are lacking. Interestingly, pDC have been shown to express the IFNλR and respond directly to IFNλ (116, 117). Whether IFNλ signaling is altered in pDC in the context of immunity is an interesting question that could have implications for immune-mediated treatment.

## IFNλ Immune Modulatory Effects

**Figure 2:**
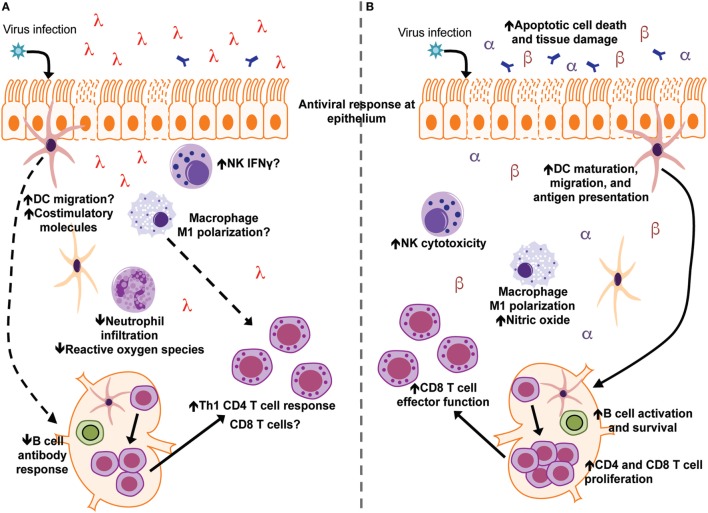
Interferon lambda (IFNλ) and IFNα/β differentially modulate immune responses during acute viral infection and tissue inflammation. **(A)** Following viral infection/tissue inflammation, IFNλ modulates functions of dendritic cells (DCs) neutrophils, CD4 T cells, and the B-cell antibody response. IFNλ signaling may also regulate macrophage, NK cell, and CD8 T cell function during infection/tissue inflammation **(B)** type I IFN (IFNα and IFNβ) have been the subject of a greater number of studies and have more defined roles during virus infection and tissue inflammation. Type I IFN enhances functions of DCs, macrophages, NK cells, B cells, CD4 T cells, and CD8 T cells toward an inflammatory/antiviral state.

The optimal induction of interferon to control infection while simultaneously avoiding host immunopathology is critical for an effective immune response against pathogens. Although IFNλ is generally considered to be less inflammatory than type I IFN, a full understanding of IFNλ’s regulation of immune responses outside of direct antiviral action has remained largely unknown (Figure 2). Recent studies, predominantly in the context of viral infections, have begun to elucidate the contribution of IFNλ to the regulation of the broader innate and adaptive immune responses (see Table 1). While the role of IFNα/β and IFNλ is similar in many viral infections, some notable differences exist. For example, therapeutic treatment of influenza virus infected mice with IFNα leads to enhanced pulmonary inflammation and mortality, while IFNλ is protective (54, 55). In addition, IFNλ is critical for protection against intestinal viral pathogens such as reovirus and rotavirus (89, 90). This is likely due to the fact that IECs respond robustly to IFNλ, but not type I IFN, *in vivo* (30). However, there are also other important roles for IFNλ in other immune cell types in the intestine, such as murine neutrophils, that have only begun to be investigated (114). As part of the ongoing efforts to understand specific function of IFNs in host defense, more studies designed to examine specific effects on different tissues and cell types that contribute to innate and adaptive immunity will be informative.

### Effects of IFN on Innate Immune Cells

IFN has been well described to have a direct antiviral effect in epithelial cells. Further, type I IFNs function on innate immune cells, such as DCs and macrophages. However, the functions of IFNλ on immune cells still remain largely unknown. For example, type I IFN signaling is well described to promote activation, survival, and cytotoxic function of NK cells during infection (118–120). By contrast, whether NK cells express IFNλR and respond directly to IFNλ to modulate NK cell function remains unclear (119, 121, 122). In this section, we will review the literature on the effect of IFNλs on innate immune cells.

#### IFN in Monocytic Cell Populations

Type I IFN promotes the polarization of macrophages to an inflammatory “M1” phenotype and increases the production of nitric oxide (73, 123–125). Type I IFN also enhances DC function by promoting their generation from monocytic precursors and leads to upregulation of MHC and costimulatory molecules in addition to increasing IL-12 production and enhancing DC migration (2, 126, 127). Conversely, the role of IFNλ in macrophages and DCs remains unresolved as studies have both supported and refuted the ability of these cells to directly respond to IFNλ (73, 128–130). Limited studies have indicated a role for IFNλ in the modulation of DC function. For example, it was demonstrated that human DC migration was enhanced as a direct response to IFNλ1 in the context of Dengue virus infection (64). In a separate report, IFNλ2 treatment increased IL-12 production and alteration of expression of the costimulatory molecule OX40L in CD11c+ cells (14). These changes, which may result in enhanced T cell immunity (131), indicate that IFNλ2 may also have a functional role at the interface of the innate and adaptive immune responses. While data implicating IFNλ regulation of DC functions are intriguing, further studies are needed to determine whether the defect in DCs in the absence of IFNλR signaling is intrinsic to these cells or influenced by the IFNλ response in epithelial cells at infection sites.

A possible explanation of the mixed reports regarding the contribution of IFN to DC function is that this could be due to differential responsiveness of various DC subsets to IFNλ and/or type I IFN. For example, pDC have been described to respond to both type I and type III IFNs to enhance their upregulation of ISGs, maturation, and antigen presentation function. We will not elaborate herein on the functions of IFN in pDC, as they have been well described in other recent reviews (132, 133). Given that there is a specific response of pDC to IFNλ that is not observed in the bulk heterogeneous DC population, it is possible other DC subsets may respond to IFNλ. During influenza and other viral infections in mice, CD103+ DCs are integral in delivery of antigen from the infected tissue to lung-draining lymph nodes where they can activate T cells (134–136). CD103+ DCs are less responsive to type I IFN, allowing for viral replication within these cells, and potentially leading to enhanced antigen presentation (137). Whether this difference could be due to preferential usage of IFNλR signaling to enhance antigen presentation has not been addressed. Interestingly, however, the ImmGen database indicates murine CD103+ DCs have higher levels of IFNλR compared with other DC subsets (138). However, as of this writing, the responsiveness of various DC and macrophage subsets to IFNλ signaling remains unclear. As T cells do not respond directly to IFNλ, it is likely that differential IFNα/β signaling compared with IFNλ in DCs could be modulating T cell responses (43, 61). Indeed, during Dengue virus infection, IFNλ leads to enhanced migration of DCs *in vitro* and increases CCR7 required for migratory function on DCs (64). Perhaps IFNλ signaling in DCs allows for optimal maturation and antigen presentation to T cells without excessive inflammation associated with IFNα/β signaling. It is also possible that at mucosal and barrier epithelial sites, epithelial cells themselves are regulating the alteration in DC response. Future studies in mice conditionally lacking IFNλR1 or IFNAR1 in DCs or epithelial cells specifically will delineate the role of IFNλ in these cell population.

#### IFN in Neutrophils

While few studies that have interrogated the direct effect of IFNα/β signaling on neutrophils, type I IFNs have been demonstrated to play a role in activation of neutrophil function (139). Murine neutrophils have recently been shown to express high levels of *Ifnlr1* and respond directly to stimulation with IFNλ (114). Treatment of mice with arthritic symptoms with IFNλ2 was shown to prevent neutrophil infiltration into arthritic joints (113). While the potential therapeutic application of IFNλ to limit neutrophil-mediated pathology is interesting in this arthritis model, whether this paradigm is true should continue to be examined in the context of other inflammatory events. Neutrophils are known to significantly exacerbate disease severity during respiratory viral and bacterial infections and directly contribute to lung pathology [reviewed in Ref. (140)]. It is intriguing that IFNλ could potentially reduce or prevent neutrophil-mediated detrimental lung inflammation during respiratory infection *via* a similar mechanism. IFNλ has recently been demonstrated to act on neutrophils to control both influenza virus infection and DSS-induced colitis in murine models, indicating IFNλ directly alters neutrophil function in addition to recruitment as previously described (35, 114, 141). Interestingly, this IFNλ-specific dampening of neutrophil function is mediated in a non-transcriptional/translational fashion *via* Akt’s regulation of the release of reactive oxygen species (114). Importantly, this study represents the first reported such function of IFNλ and opens the intriguing possibility for IFNλ to yield changes in immune cells in a mechanism distinct from canonical JAK–STAT signaling. While these studies are intriguing, they have thus far only been validated in murine neutrophils. Future studies will be needed to determine whether human neutrophils respond to IFNλ in a similar fashion.

### Effects of IFNs on Adaptive Immune Cells

Adaptive immunity is critical in controlling and providing long-term protection against infection. IFNs act at the interface of innate and adaptive immunity, by directly regulating innate as well as adaptive immune cells. For example, type I IFN promotes B-cell activation and class switching during acute viral infection [reviewed in Ref. (2)]. While there is currently no evidence demonstrating IFNλ has direct effects on the function of B cells, humans receiving influenza virus vaccination who had lower levels of circulating IFNλ correlated with increased sero-conversion (15). In addition, IFNλ has been reported to augment TLR-mediated activation and function of human B cells, but IFNλ could not directly and independently impact B-cell activation (142). Conversely, in a murine model of WNV infection, IFNLR1−/− mice had no effect on antibody responses compared with wild-type control mice (84). However, evidence supporting a role for IFNλ regulation of B cell functions is currently lacking but this still an area of active investigation.

#### IFN in T Cells

While T cells do not respond directly to IFNλ (43, 61), it is clear that IFNλ regulates function of T cells. IFNλ enhances T cell proliferation and Th1/Th17 cytokine production following treatment of peripheral blood mononuclear cells with IFNλ and in the context of asthma and influenza virus vaccination (14, 15). IFNλ has been shown to polarize the response toward a Th1 phenotype while suppressing Th2 and associated B cell responses. This is supported by studies in humans evaluating a SNP (rs8099917) in the *IFNL* locus, where individuals with the SNP that correlate to high IFNλ3 levels have lower sero-conversion rates following influenza virus vaccination, but a greater induction of Th1 CD4 T cells (15). Therefore, IFNλ-mediated effects on the T cell response might be indirect mediated by another cell subset known to express IFNλR. It is likely that IFNλ signaling in DCs is responsible for this alteration of the T cell response; however, the direct action of DCs in regulating Th1/Th2 responses is still unknown. In addition, whether IFNλ alters DCs to regulate CD8 T cell responses, which are critical for clearance of virus during many infections, is still unknown. Intriguingly, a report investigating acute and chronic LCMV responses in a murine model suggest IFNλ signaling negatively regulates virus-specific T cell responses during acute infection, but is required for the persistence of the T cell response during chronic infection (61). While the mechanism remains unclear, a study in macaques demonstrated that IFNλ3 drives cytotoxic ability of CD8 T cells, overall providing further evidence of the potential of IFNλ to function as an immune adjuvant or therapeutic agent to promote antiviral T cell responses (143).

In contrast to the absence of direct effects by IFNλ on CD4 and CD8 T cell activation, proliferation, and cytokine production, type I IFN directly regulates these T cell functions [reviewed in Ref. (144)]. Type I IFN signaling can regulate T cell responses *via* indirect effects on DCs or macrophages in addition to direct signaling effects on T cells themselves. This difference in mechanisms of T cell regulation is a major distinction between type I and type III IFNs that has not yet been fully evaluated. The potentially distinct, indirect mechanism of IFNλ regulation of T cell responses could yield interesting insights into the ability of IFNλ to be utilized as a therapeutic or vaccine adjuvant to augment the immune response against viral infections.

## Role of Type III IFNs at the BBB

In addition to impacts on immune cells, IFNλ has also been described to regulate the BBB during WNV infection (84). Mice lacking the IFNλR1 show increased viral titers in central nervous system tissues and increased BBB permeability following WNV infection. Interestingly, IFNλ-mediated restriction of the endothelial tight junctions in an *in vitro* BBB model is independent of STAT1 or protein synthesis. These findings suggest there may be an undescribed, novel IFNλ signaling pathway that regulates endothelial cells. As endothelial cells are significant regulators of inflammatory responses, IFNλ could exert important effects on these cells types that would also be applicable to infection at the site of pulmonary and gastrointestinal barriers.

## Outstanding Questions

While the functions of IFNλ and IFNα/β overlap in many infections and cell types, a growing number of notable differences are allowing for a better understanding of specialized roles of IFNs in regulation of immunity. In addition, the difference in IFNAR and IFNλR1 expression levels on various cell subsets and tissues could contribute to specific action of IFNλ vs type I IFN. This regulation along with SNPs in *IFNL* genes highlight that IFNλ has a unique role in antiviral immunity independent of type I IFN. While nearly 15 years of research has led to many insights in the function of IFNλ and its contribution to immunity, many questions remain to be answered. What are the distinct and redundant functions of each *IFNL* gene? Are there spatiotemporal effects that provide distinctions of IFNλ subtypes? Are there other signaling pathways that are active downstream of IFNλR differentially activate immune and epithelial cell subsets to modulate innate and adaptive immune response? Answering these questions with the help of newly available murine models will be critical to gain insights into the function of IFNλ, and to continue to develop IFNλ for use as a therapeutic against viral infections in the liver and at barrier surfaces.

## Author Contributions

All authors contributed to the conceptualization, writing, and editing of the manuscript.

## Conflict of Interest Statement

The authors declare that the research was conducted in the absence of any commercial or financial relationships that could be construed as a potential conflict of interest.
